# Supplementary material for: Differential Photosensitivity of Fibroblasts Obtained from Normal Skin and Hypertrophic Scar Tissues
Source: Int J Mol Sci. 2024 Feb 9;25(4):2126. doi: 10.3390/ijms25042126 (PMC10889571; doi:10.3390/ijms25042126)
Supplement: Supplementary file 1 [file ijms-25-02126-s001.zip › ijms-2821806-supplementary.docx]

**Supplementary Materials**

Differential Photosensitivity of Fibroblasts Obtained from Normal Skin and Hypertrophic Scar Tissues

Junya Kusumoto ^1,2,^*, Masaya Akashi ^2^, Hiroto Terashi ^1^ and Shunsuke Sakakibara ^1^

^1^ Department of Plastic Surgery, Kobe University Graduate School of Medicine, Kobe 650-0017, Japan; terashi@med.kobe-u.ac.jp (H.T.); shunsuke@med.kobe-u.ac.jp (S.S.)

^2^ Department of Oral and Maxillofacial Surgery, Kobe University Graduate School of Medicine,
Kobe 650-0017, Japan; akashim@med.kobe-u.ac.jp

***** Correspondence: chivalry_2727@people.kobe-u.ac.jp; Tel.: +81-78-382-6213; Fax: +81-78-382-6229

1. Supplementary Methods

1.1. Reverse transcription PCR (RT-PCR)

RT-PCR analysis of the expression levels of opsins was performed using the total RNA isolated from primary cultured cells using a PrimeScript One Step RT-PCR kit Ver.2 (Takara Bio, Shiga, Japan). Briefly, RT-PCR was performed by mixing enzymes (including reverse transcriptases, Hot Start Taq DNA polymerase, and RNase inhibitors), a buffer, dNTP mixtures, a forward primer (5’-GGTCATGGTCACCTGGTCACTCCAACAATATC-3’; 20 μM), a reverse primer (CCTGTCCCCATCTTTCTGTGACATCACAATGG-3’; 20 μM), total RNA, and distilled water. The PCR conditions were as follows: cDNA synthesis at 50 °C for 30 min; thermal denaturation at 94 °C for 2 min; 35 cycles of thermal denaturation at 94 °C for 30 s; annealing at 55.3 °C for 20 s; extension at 72 °C for 30 s; and final extension at 72 °C for 7 min (Takara PCR Thermal Cycler Dice Version TP600/Tp650). The PCR products were sequenced.

1.2. real-time quantitative reverse transcription PCR (qRT-PCR)

Quantitative analysis of the mRNA expression of opsins was performed via qRT-PCR using total RNA samples recovered from human skin tissue. β-actin was used as an endogenous control. The qRT-PCR results were analyzed using the ΔΔC_t_ method along with TaqMan probes; the analysis was performed following the protocol for the One Step PrimeScript RT-PCR Kit (Perfect Real Time) (Takara Bio).

Supplementary Figures


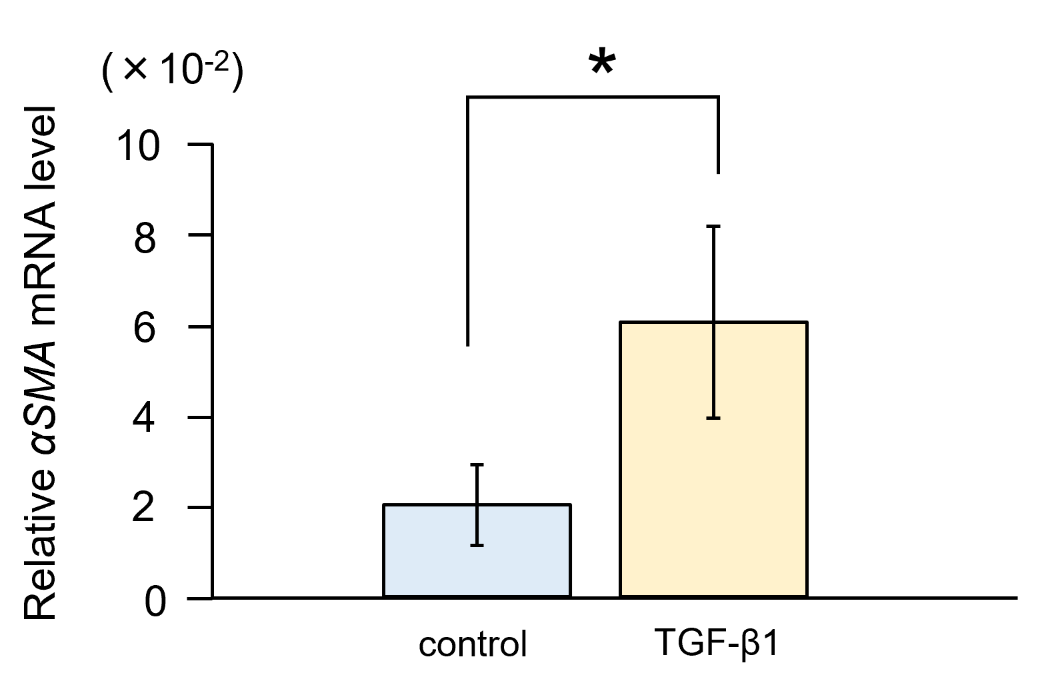


**Figure S1.** Effect of TGF-β1 on fibroblasts derived from normal skin tissue.

The addition of TGF-β1 (10 ng/mL) to normal dermal fibroblasts (NDFs) significantly increased the gene expression level of αSMA (n = 4; Student's t-test; p = 0.013). The levels of αSMA in NDFs were comparable to those in hypertrophic scar fibroblasts (HSF) (p = 0.375).


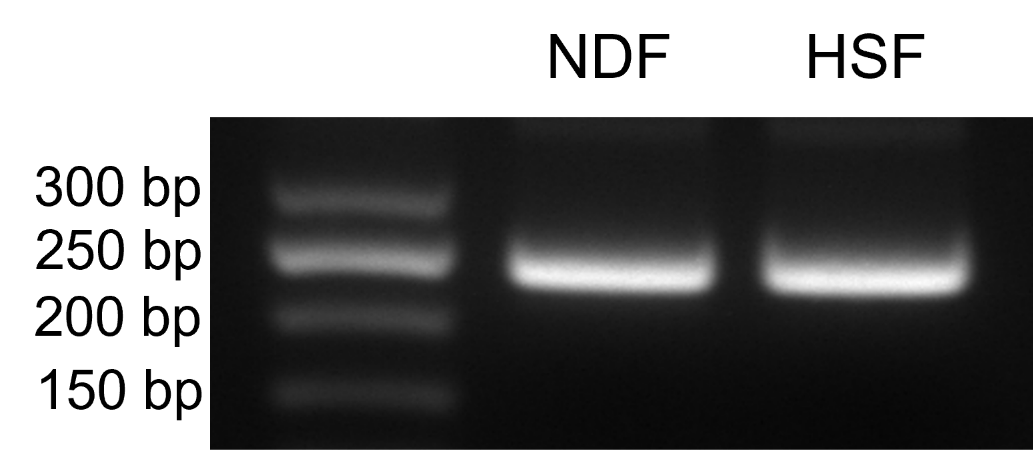


**Figure S2.** Expression of *OPN3* in cultured cells.

From the left: marker, NDFs, and HSFs. Primers F and R amplified a band in the expected size range of approximately 237 bp.


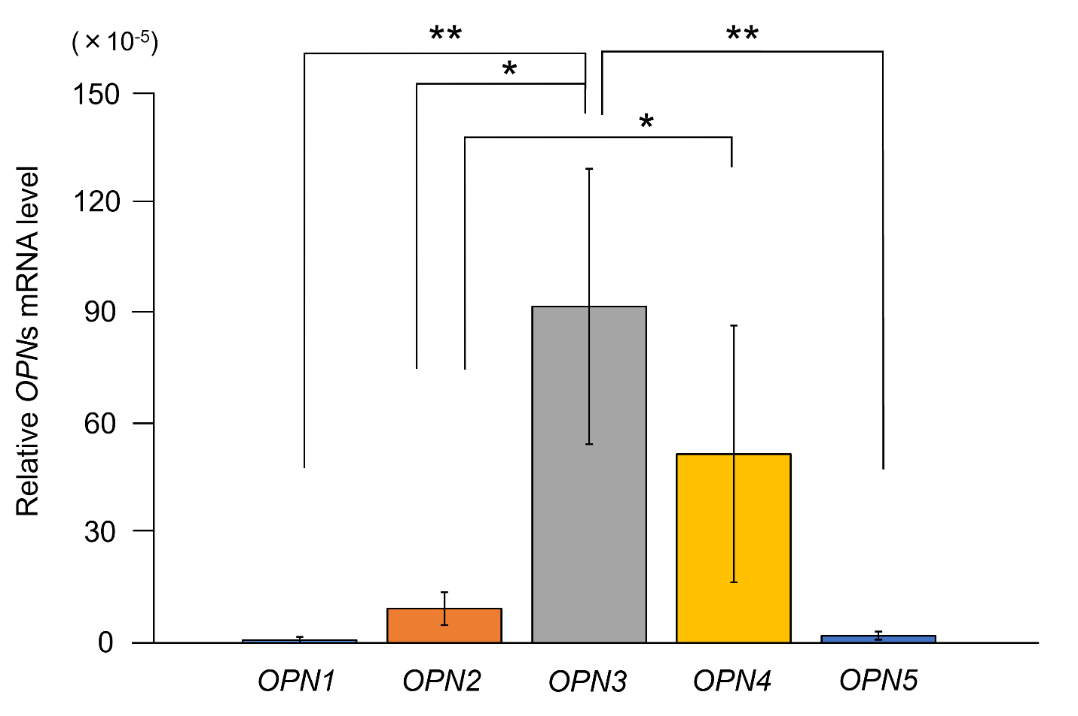


**Figure S3.** Gene expression levels of opsins in normal skin tissue.

In normal skin tissues, as in cultured cells, the expression of *OPN3* was the highest among all opsins. In contrast, the expression level of *OPN4* in normal skin tissues was higher than in cultured cells and comparable to that of *OPN3* (n = 4; relative to the mean of the expression level referred to β-actin; Tukey’s test, * p < 0.05, ** p < 0.001).


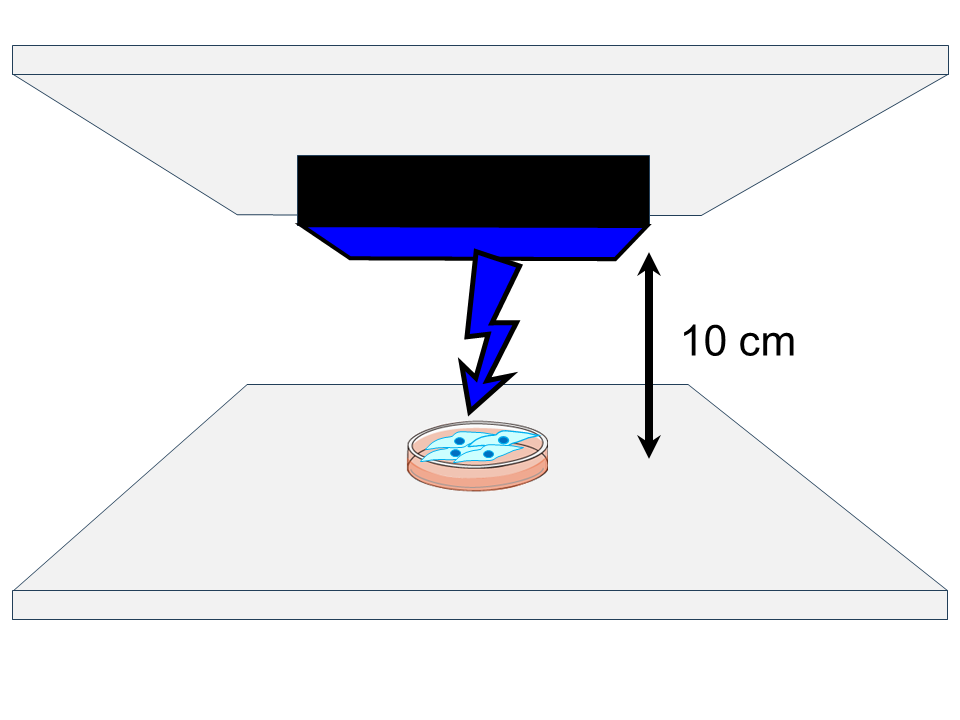


**Figure S4.** Blue light irradiation device and conditions.

The blue light irradiation device was inserted in a CO2 incubator and blue light was irradiated under dark conditions. The distance between the light source and cultured cells was 10 cm, and the irradiation dose was 8.0 W/m^2^ for 10 min (0.5 J/cm^2^).
